# Current sample size conventions: Flaws, harms, and alternatives

**DOI:** 10.1186/1741-7015-8-17

**Published:** 2010-03-22

**Authors:** Peter Bacchetti

**Affiliations:** 1 Department of Epidemiology and Biostatistics, Box 0560, University of California, San Francisco, CA 94143, USA

## Abstract

**Background:**

The belief remains widespread that medical research studies must have statistical power of at least 80% in order to be scientifically sound, and peer reviewers often question whether power is high enough.

**Discussion:**

This requirement and the methods for meeting it have severe flaws. Notably, the true nature of how sample size influences a study's projected scientific or practical value precludes any meaningful blanket designation of <80% power as "inadequate". In addition, standard calculations are inherently unreliable, and focusing only on power neglects a completed study's most important results: estimates and confidence intervals. Current conventions harm the research process in many ways: promoting misinterpretation of completed studies, eroding scientific integrity, giving reviewers arbitrary power, inhibiting innovation, perverting ethical standards, wasting effort, and wasting money. Medical research would benefit from alternative approaches, including established *value of information *methods, simple choices based on cost or feasibility that have recently been justified, sensitivity analyses that examine a meaningful array of possible findings, and following previous analogous studies. To promote more rational approaches, research training should cover the issues presented here, peer reviewers should be extremely careful before raising issues of "inadequate" sample size, and reports of completed studies should not discuss power.

**Summary:**

Common conventions and expectations concerning sample size are deeply flawed, cause serious harm to the research process, and should be replaced by more rational alternatives.

## Background

Early in my career, an epidemiologist told me that dealing with sample size is the price one has to pay for being a biostatistician. Since then, I have spent untold time and effort paying this price, while also coming to realize that such effort produces no real scientific benefit. Unfortunately, widespread misconceptions about sample size hurt not only statisticians, but also the quality of medical science generally.

The conventional expectation is that a study must have at least 80% power or else be considered scientifically unsound and even unethical [1]. Some challenges to this dogma have been based on the idea that some information is better than none and that even a small amount of inconclusive information may contribute to a later systematic review [2-4], but conventions remain entrenched and failing to anticipate systematic reviews is only one aspect of only one of three fundamental flaws. I present here a wider challenge to current conventions, including how they cause serious harm. Alternatives could produce both better studies and fairer peer review of proposed studies.

## Discussion

### Flaws in current conventions

The standard approach is based on statistical hypothesis testing where one rejects a null hypothesis of no difference if the *P*-value is < 0.05. One calculates sample size based on a specified difference of interest, an assumption about the standard deviation or event rate of the outcome being studied, and conventional choices for Type I error (chance of rejecting the null hypothesis if it is true) and statistical power (chance of rejecting the null hypothesis if the specified difference actually exists). Type I error is essentially always set to be 0.05, and sample sizes producing power less than 80% are considered inadequate. Three crucial flaws in this standard approach are that it 1) assumes a meaningful boundary between adequate and inadequate sample sizes that does not actually exist, not even approximately; 2) relies strongly on inputs that generally cannot be accurately specified; and 3) does not reflect how a completed study's information should actually be used. Although the first is perhaps the most fundamental, all three are severe, and they intertwine to produce many of the harms discussed in the next section.

Experience with previous related papers suggests that many readers will immediately formulate objections or counterarguments. Anticipating and pre-empting all these is not possible, but I comment on two of the more likely ones in Additional file 1.

#### The threshold myth

**Figure 1:**
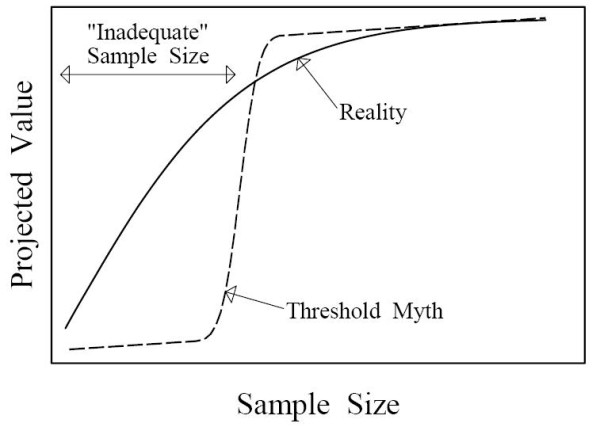
**Qualitative depiction of how sample size influences a study's projected scientific and/or practical value**. A threshold shaped relationship (dashed line) would create a meaningful distinction between adequate and inadequate sample sizes, but such a relation does not exist. The reality (solid line) is qualitatively different, exhibiting diminishing marginal returns. Under the threshold myth, cutting a sample size in half could easily change a valuable study into an inadequate one, but in reality such a cut will always preserve *more *than half of the projected value.

Current conventions hinge on an implicit assumption that there is a meaningful demarcation between adequate and inadequate sample sizes, and that having an inadequate sample size is fatal. I find this consistently reflected in the language used by my clients, collaborators, and colleagues: asking how many will be "needed to answer the question", wanting to ensure they will "have enough subjects," and calling studies with <80% power "doomed" and therefore wasteful and unethical. A statement from a recent grant review is very typical in presuming that too small a sample size could completely ruin the study: "it is unclear if the study will be sufficiently powered to allow the proposed analyses." If there were an approximately threshold-shaped relationship between sample size and the scientific or practical value that a study can be projected to produce, as shown in Figure 1, then this implicit assumption would be reasonable: falling short of the threshold would indeed result in an inadequate study. Such a shape is also needed to justify the practice of ignoring costs when setting sample size: if the correct side of the threshold were always where 80% power is produced, then current methods would automatically produce a good cost-benefit tradeoff without explicitly considering costs. Unfortunately for the standard approach, the real relationship is radically different from a threshold, instead having a concave shape that continually flattens, reflecting diminishing marginal returns. This characteristic shape was recently verified for a wide variety of measures of projected value that have been proposed for use in sample size planning, including power [5]. Falling short of any particular arbitrary goal, notably 80% power, is therefore not the calamity presumed by conventional thinking. The lack of any threshold undercuts the foundation of current standards: they guard against a non-existent danger.

#### Inherent inaccuracy

Although precise mathematical formulas are available for calculating sample sizes, these depend on specifying exact values for inputs, and changes in these inputs produce magnified changes in the resulting sample size. In particular, studies with a continuous primary outcome measure, such as quality of life score, change in telomere length, weight loss, and so on, must specify its standard deviation. This is difficult to do accurately, unless there is so much preliminary data that the study isn't really needed, but it has a big influence on sample size: a two-fold increase in the assumed standard deviation produces a four-fold increase in sample size. A four-fold larger sample size also results from halving the difference of interest (the *alternative hypothesis*). This is particularly hard to specify because even the theoretical principles for choosing it are unclear. Some assert that it should be the smallest difference that would be important to patients or for scientific or public health purposes [1], but this is often subjective and difficult to specify (what difference in survival rates is unimportant), importance is rarely all-or-nothing, and very small differences may be important, leading to impractical sample sizes. Investigators frequently use the difference suggested by preliminary data, but this is unreliable [6] and has greater risk of inconclusive results for differences that are smaller but still interesting. A case has even been made for using the difference hoped for by patients [7].

Inaccuracy of sample size calculations is not only theoretically inevitable [6,8] but also empirically verified. One careful study of assumed standard deviations in a seemingly best-case scenario, randomized trials published in four leading general medical journals, found that about a quarter had more than five-fold inaccuracy in sample size and more than half had more than two-fold inaccuracy [9]. Another study recently found high rates of inaccuracy along with many other problems [10]. This problem cannot be solved by simply trying harder to pinpoint where 80% power will be achieved, because inaccuracy is inherent in the conventional framework.

#### Design-use mismatch

Why design a study as if the only thing you will examine when it is done is whether or not *P *< 0.05? Good statistical practice demands consideration of the particular *P*-value obtained and, more importantly, of estimates and confidence intervals that quantify the effects or associations of interest [11,12]. Using a *P*-value cutoff to define a study as positive or negative was proposed as a framework for using a single study in isolation to automatically make a decision about what to do [13], but this framework is rarely actually followed in medical research. Even pivotal randomized drug trials must provide convincing evidence, not just an automatic decision: regulators would never settle for knowing nothing about a study's results other than *P *< 0.05. In addition, much of the value of an individual study may derive from its contribution to later systematic reviews [2-4], which will not make any use of the study's *P*-value. Planning for a use that will not (or should not) occur cannot be expected to optimize a study's design. (Importantly, the threshold myth and inherent inaccuracy remain severe problems even for the rare cases where a study is likely to be the sole basis for making an automatic decision based only on whether *P *< 0.05.)

### Harms from current conventions

Because of the reality illustrated in Figure 1, the choice of sample size is less important than generally supposed. Much of the substantial harm from current conventions therefore results not from bad sample size choices but from unrealistic expectations and strict enforcement of misguided standards.

#### False assurance and promotion of misinterpretation

The idea that a carefully chosen "adequate" sample size can ensure that a study will be definitive, reflected in the common phrase "enough to answer the question", is certainly appealing, but it is just wishful thinking. In addition to inherent inaccuracy and the fact that an 80% chance is far from a certainty, even huge studies can produce results near the boundary of what is large enough to be important. For example, a mega-study of the influence of dietary fat intake on breast cancer risk [14] produced an estimated effect part way between what had been expected and no effect. Far from ensuring that a study will be definitive, claims of 80% power actually provide no information about how valuable any particular study is likely to be, because every proposal contains such a claim.

False assurance leads directly to the following logic: "Sample size is adequate to ensure a definitive result, the result is not definitively positive (that is, *P *> 0.05), therefore the result is definitively negative." I have encountered many researchers who believe this logic, and the widespread practice of considering power when interpreting so-called negative studies [15] seems aimed at determining when this reasoning can be applied. This resolves the design-use mismatch, but in the wrong way, by focusing only on whether *P *< 0.05. Although investigators usually report estimates, confidence intervals, and attained *P*-values, they often ignore these very informative results when interpreting their studies. For example, a study of vitamin C and E supplementation in pregnancy reported rates of infant death or other serious outcomes that implied one outcome prevented for every 39 women treated [16]. The authors nevertheless concluded definitively that supplementation "does not reduce the rate", because the *P*-value was 0.07. Interpreting *P *> 0.05 as indicating that the results actually observed must be an illusion is very poor reasoning, but I find it in most draft manuscripts I review and many published articles I read. Interpretation of *P *< 0.05 as ensuring that an observed effect is real and accurate also seems to be widespread, despite being unreliable [17].

#### Erosion of scientific integrity

Manipulation of sample size calculations to produce a desired result is a well-acknowledged phenomenon [3,18,19] that has been described as a "sample size game" [20] or "sample size samba" [21]. A published dramatization of the process ends with the statistician asking a client, "Where do you want to start fudging?" [22] Changing the specified difference of interest makes such manipulation easy, and unavoidable practical realities often make it necessary, either because cost or feasibility constraints cannot be exceeded or because there is not enough information about inputs to permit a meaningful calculation. Sample size is then chosen based on other criteria, but it must still be justified in terms of power. Forcing investigators to hide the real reason for choosing a sample size sends a bad message about integrity, right at the beginning of the research process.

#### Arbitrary reviewer power

Because of the strong reliance on uncertain inputs, any reviewer who is so inclined can question the assumptions and results of any power calculation. A minor change in the inputs can indicate that the proposed sample size falls substantially short of "adequate". Whether a sample size justification passes peer review therefore depends on arbitrary reviewer discretion, which is a bad situation for a process meant to be fair. Because criticism of sample size plans is always possible and very common [23], it is ideal for providing cover when reviewers cannot pinpoint, or are unwilling to admit, the real reasons why they dislike a proposal.

#### Barrier to innovation

Proposals to evaluate new ideas or issues face particular difficulty, because the lack of adequate knowledge about inputs for standard calculations is especially obvious. Although a National Institutes of Health task force on peer review argued that no preliminary data should be required for new ideas [24], this does not solve the problem of justifying a sample size. Innovators must usually rely on guesses that are obviously arbitrary or provide calculations based on standardized effect sizes that have no real connection to the study being proposed. This leaves them especially vulnerable to the arbitrary reviewer power noted above.

#### Wrong-way ethical standards

The contention that inadequate power makes a study unethical [1] relies entirely on the threshold myth, a false belief that studies with less than 80% power cannot be expected to produce enough scientific or practical value to justify the burden imposed on participants. Because larger studies burden more participants, the fact of diminishing marginal returns implies that the ratio of projected value to total participant burden can only get worse with larger sample sizes. The risk of inadequate projected value relative to participant burden therefore applies to studies that are too large, not too small [25-27].

#### Wasted effort

Because conventional sample size planning produces no real benefit, it wastes time and talent. Investigators often struggle to understand what is needed for calculations, to find even semi-relevant preliminary data, and to somehow formulate and justify seemingly arbitrary guesses and choices. Statisticians often help with all of the above, determine appropriate simplifications and approximations, and painstakingly piece together relevant inputs from published graphs or partial information. This reduces time and effort available for producing real scientific value.

#### Wasted money

Without the threshold myth, it makes no sense to set a sample size goal that must be reached regardless of cost, but cost has no role in the standard approach. Ignoring costs is so impractical that investigators may often take them into account. Actually following the conventional approach can produce severely cost-inefficient sample size choices [5]. In addition, the threshold myth promotes a default strategy of concentrating more resources in fewer, larger studies [28]. Such concentration can be efficient if the funded studies are much more promising than their competitors [29], but concentration will be inefficient whenever there are many possible studies with similar promise or when reviewers nix small but cost-efficient studies for having supposedly inadequate sample sizes. Concentration may be particularly poor when allocating limited patients among competing studies, because adding more patients to an already-large study not only produces less and less incremental value but also takes away more and more potential value from other studies: diminishing marginal returns imply increasing marginal opportunity costs.

### Alternatives

Abandoning the 80% power standard need not lead to sample size nihilism, where "we abandon the current delusion that sample size matters" [3]. There are methods for making sensible sample size decisions while avoiding the drawbacks of current conventions.

#### Value of information methods

Many methods have already been described in the statistical literature for choosing the sample size that maximizes the expected value of the information produced minus the total cost of the study. See [18] for an early discussion, [30,31] for recent examples, and the introduction of [5] for additional references. These require projecting both value and cost at various different sample sizes, including quantifying cost and value on the same scale (note, however, that this could be avoided by instead maximizing value *divided by *total cost). They also require formally specifying uncertainty about the state of nature; although this can be criticized as being subjective, it improves vastly on the usual conventional approach of assuming that one particular guess is accurate. These methods can require considerable effort and technical expertise, but they can also produce the sort of thorough and directly meaningful assessment that should be required to justify studies that are very expensive or that put many people at risk.

#### Simple choices based on cost or feasibility

Recent work has justified two simple choices that are based only on costs [5], with no need to quantify projected value or current uncertainty about the topic being studied. Because costs can generally be more accurately projected than the inputs for conventional calculations, this avoids the inherent inaccuracy that besets the conventional approach. One choice, called *n*_min_, is the sample size that minimizes the total cost per subject studied. This is guaranteed to be more cost-efficient (produce a better ratio of projected value to cost) than any larger sample size. It therefore cannot be validly criticized as inadequate. The other, called *n*_root_, is the sample size that minimizes the total cost divided by the square root of sample size. This is smaller than *n*_min _and is most justifiable for innovative studies where very little is already known about the issue to be studied, in which case it is also guaranteed to be more cost efficient than any larger sample size. An interactive spreadsheet that facilitates identification of *n*_min _and *n*_root _is provided as Additional file 2.

A common pragmatic strategy is to use the maximum sample size that is reasonably feasible. When sample size is constrained by cost barriers, such as exhausting the pool of the most easily studied subjects, this strategy may closely approximate use of *n*_min _and therefore share its justification. When constraints imposed by funders determine feasibility, doing the maximum possible within those constraints is a sensible choice.

#### Sensitivity analysis

Sample size planning involves considerable uncertainty, and a simple and familiar way of assessing uncertainty is with sensitivity analyses: examining how results change under different assumptions. I propose a framework, illustrated in Table 1, for presenting a useful array of possibilities for a study's most important products, the estimated effect and its confidence interval. This is based on varying 1) assumptions that determine the precision of estimates and 2) the observed effect size. Together with discussion of the potential value of each resulting outcome, this provides an informal assessment of the value of the information that may result. This is less systematic than the value of information methods mentioned above, but it covers a range of likely scenarios, avoids technical difficulties of the mathematically formalized methods, and focuses on particular concrete results, which allows reviewers to easily assess the claimed potential value. Because the table entries show the information that would be used in a systematic review, the potential value can be discussed in terms of how it would modify a recent review or contribute to future ones, if those are deemed to be the most important considerations.

**Table 1:** Sample layout of sensitivity analysis.

		**^Box# ^ Observed difference in outcome rates (95% CI)**		
		
		**Expected** **-2.8%**	**Intermediate** **-1.4%**	**Null** **0%**
		
Observed	Low 3%	^1 ^ -2.8 (-3.9 to -1.6)	^2 ^ -1.4 (-2.7 to -0.03)	^3 ^ 0 (-1.5 to +1.5)
	
outcome rate	Expected 6.5%	^4 ^ -2.8 (-4.8 to -0.8)	^5 ^ -1.4 (-3.5 to +0.7)	^6 ^ 0 (-2.2 to +2.2)
	
in controls	High 12%	^7 ^ -2.8 (-5.6 to +0.01)	^8 ^ -1.4 (-4.3 to +1.5)	^9 ^ 0 (-2.9 to +2.9)

Shown are possible study results with a given sample size (935 per group, based on the vitamin study discussed above [16]), for a yes or no outcome. Rows have differing assumptions concerning precision of the estimates, ranging from high precision (top row) to low precision (bottom row). For a continuous outcome, the rows would instead be for optimistic (small), expected, and pessimistic (large) standard deviations.

The entries in the table are exactly the key results that interpretation should focus on when the study is completed, so this properly aligns planning with eventual use. The middle row can be a best guess such as would be used for conventional calculations; the other rows should reflect a reasonable range of uncertainty, which will depend on what is already known about the topic being studied. For the columns, inclusion of the intermediate case is important, because this will often include the most problematic or disappointing potential results. The vitamin study [16] paired a safe and inexpensive intervention with a severe outcome, so even results in the middle column would be regarded as encouraging; the actual completed study landed essentially in Box 7, which should have been interpreted as very encouraging even though not definitive. Boxes 8 and 9 will usually be the least useful, but as noted above (False assurance), the risk of disappointing results is always present and should not be considered a flaw in study design.

#### Previous similar or analogous studies

A simple way to choose a sample size is to use one that has worked well in the past for similar or analogous studies. Because exactly relevant preliminary data are often unavailable, assumptions for power calculations are frequently based on such studies, anyway. Skipping the over-formalized and inherently unstable power calculation process and just using the previous sample size may be a reasonable approach.

### Getting there from here

The culture around sample size planning seems to be extraordinarily entrenched, so change may be difficult. Nevertheless, the following actions could help move medical research toward more rational expectations.

Research training should not present current conventions as unquestionable dogma. Although trainees must know about the culture they will have to face, education about sample size should be balanced. For example, this article could be discussed.

When preparing a study proposal, courageous investigators could use an alternative approach from the previous section. This may be most practical for highly innovative proposals where standard power calculations would most clearly be meaningless. For other studies, use of detailed value of information methods may be convincing when the effort they require can be devoted. In many cases, it may be safer to supplement standard power calculations with more meaningful reasoning regarding sample size. This avoids dishonesty and at least gives reviewers the option of focusing on what really matters. Also, the juxtaposition of standard and alternative reasoning may help promote recognition of the standard approach's inadequacies.

Stemming criticism of sample size in the peer review process is necessary to allow alternative approaches to take hold. Reviewers should usually refrain from criticizing sample size and should challenge fellow reviewers who do. If fellow reviewers feel that a study is only half as large as it should be, remind them that this does not mean that the study is doomed to be worthless; instead, it will have *more *than half the projected value that it would with the doubled size. Sample size criticism is currently too easy and convenient; challenging fellow reviewers will help to change this.

Reports of completed studies should not include power calculations, and guidelines requiring them [11] should be changed to instead discourage them. Reporting power calculations has been justified as a way to disclose the primary outcome and the original target sample size [21,32], but these can be stated directly without any reference to a power calculation [33]. Because power calculations are often not the real reason for the chosen sample size, providing them for completed studies does not promote, but rather subverts, full, transparent reporting. In addition, power is irrelevant for interpreting completed studies [15,20,34,35], because estimates and confidence intervals allow more direct and reliable interpretation. Reporting power calculations inevitably gives the impression that they matter for interpretation, which serves to reinforce the widespread misconception that they allow any result with *P *> 0.05 to be interpreted as proving the null hypothesis [33].

## Summary

The status quo concerning sample size is unacceptable because of severe inherent flaws and substantial harm to the research process. Perhaps most notably, the threshold myth is clearly unrealistic, but it is an essential underpinning of the common and pernicious practice of condemning studies thought to be "underpowered". Despite lack of any valid rationale or supporting evidence, current conventions are so deeply entrenched and widely enforced that inertia alone may perpetuate them for some time. I encourage any who are persuaded by the case presented here to take action to hasten reform.

## Competing interests

The author declares that he has no competing interests.

## Pre-publication history

The pre-publication history for this paper can be accessed here:

http://www.biomedcentral.com/1741-7015/8/17/prepub

## Supplementary Material

Additional file 1 **Comments on two possible objections**. Discusses two possible objections to the case made in this paper. Click here for file

Additional file 2 **Cost-based sample size**. This is an interactive Microsoft Excel spreadsheet that facilitates determination of sample sizes *n*_min _and *n*_root _using the simple cost-based methods noted in the Alternatives section. Click here for file

## References

[B1] Halpern SD Karlawish JHT Berlin JA The continuing unethical conduct of underpowered clinical trials JAMA-Journal of the American Medical Association 2002 288 358 362 10.1001/jama.288.3.358 12117401

[B2] Edwards SJL Lilford RJ Braunholtz D Jackson J Why "underpowered" trials are not necessarily unethical Lancet 1997 350 804 807 10.1016/S0140-6736(97)02290-3 9298015

[B3] Guyatt GH Mills EJ Elbourne D In the era of systematic reviews, does the size of an individual trial still matter? PLoS Medicine 2008 5 3 5 10.1371/journal.pmed.0050004 PMC2174963 18177203

[B4] Vail A Experiences of a biostatistician on a UK research ethics committee Statistics in Medicine 1998 17 2811 2814 10.1002/(SICI)1097-0258(19981230)17:24<2811::AID-SIM22>3.0.CO;2-1 9921603

[B5] Bacchetti P McCulloch CE Segal MR Simple, defensible sample sizes based on cost efficiency Biometrics 2008 64 577 585 10.1111/j.1541-0420.2008.01004_1.x 18482055 PMC2769573

[B6] Kraemer HC Mintz J Noda A Tinklenberg J Yesavage JA Caution regarding the use of pilot studies to guide power calculations for study proposals Archives of General Psychiatry 2006 63 484 489 10.1001/archpsyc.63.5.484 16651505

[B7] Horrobin DF Are large clinical trials in rapidly lethal diseases usually unethical? Lancet 2003 361 695 697 10.1016/S0140-6736(03)12571-8 12611394

[B8] Matthews JNS Small clinical-trials - are they all bad? Statistics in Medicine 1995 14 115 126 10.1002/sim.4780140204 7754260

[B9] Vickers AJ Underpowering in randomized trials reporting a sample size calculation Journal of Clinical Epidemiology 2003 56 717 720 10.1016/S0895-4356(03)00141-0 12954462

[B10] Charles P Giraudeau B Dechartres A Baron G Ravaud P Reporting of sample size calculation in randomised controlled trials: review British Medical Journal 2009 338 b1732 10.1136/bmj.b1732 19435763 PMC2680945

[B11] Altman DG Schulz KF Moher D Egger M Davidoff F Elbourne D Gotzsche PC Lang T Grp C The revised CONSORT statement for reporting randomized trials: Explanation and elaboration Annals of Internal Medicine 2001 134 663 694 11304107 10.7326/0003-4819-134-8-200104170-00012

[B12] Gardner MJ Altman DG Confidence-intervals rather than P-values - estimation rather than hypothesis-testing British Medical Journal 1986 292 746 750 10.1136/bmj.292.6522.746 3082422 PMC1339793

[B13] Goodman SN P-values, hypothesis tests, and likelihood - implications for epidemiology of a neglected historical debate American Journal of Epidemiology 1993 137 485 496 8465801 10.1093/oxfordjournals.aje.a116700

[B14] Prentice RL Caan B Chlebowski RT Patterson R Kuller LH Ockene JK Margolis KL Limacher MC Manson JE Parker LM Paskett E Phillips L Robbins J Rossouw JE Sarto GE Shikany JM Stefanick ML Thomson CA Van Horn L Vitolins MZ Wactawski-Wende J Wallace RB Wassertheil-Smoller S Whitlock E Yano K Adams-Campbell L Anderson GL Assaf AR Beresford SA Black HR Low-fat dietary pattern and risk of invasive breast cancer - The women's health initiative randomized controlled dietary modification trial JAMA-Journal of the American Medical Association 2006 295 629 642 10.1001/jama.295.6.629 16467232

[B15] Hoenig JM Heisey DM The abuse of power: The pervasive fallacy of power calculations for data analysis American Statistician 2001 55 19 24 10.1198/000313001300339897

[B16] Rumbold AR Crowther CA Haslam RR Dekker GA Robinson JS Vitamins C and E and the risks of preeclampsia and perinatal complications New England Journal of Medicine 2006 354 1796 1806 10.1056/NEJMoa054186 16641396

[B17] Ioannidis JPA Why most published research findings are false PLoS Med 2005 2 8 e124 http://www.plosmedicine.org/article/info%3Adoi%2F10.1371%2Fjournal.pmed.0020124 16060722 10.1371/journal.pmed.0020124 PMC1182327

[B18] Detsky AS Using cost-effectiveness analysis to improve the efficiency of allocating funds to clinical-trials Statistics in Medicine 1990 9 173 184 10.1002/sim.4780090124 2111932

[B19] Senn S Statistical Issues in Drug Development 2007 2 Chichester, England; Hoboken, NJ: John Wiley & Sons

[B20] Goodman SN Berlin JA The use of predicted confidence-intervals when planning experiments and the misuse of power when interpreting results Annals of Internal Medicine 1994 121 200 206 8017747 10.7326/0003-4819-121-3-199408010-00008

[B21] Schulz KF Grimes DA Epidemiology 1 - Sample size calculations in randomised trials: mandatory and mystical Lancet 2005 365 1348 1353 10.1016/S0140-6736(05)61034-3 15823387

[B22] Norman GR Streiner DL PDQ Statistics 2003 3 Hamilton, Ont.: B.C. Decker

[B23] Bacchetti P Peer review of statistics in medical research: the other problem British Medical Journal 2002 324 1271 1273 10.1136/bmj.324.7348.1271 12028986 PMC1123222

[B24] Panel On Scientific Boundaries For Review Recommendations for change at the NIH's center for scientific review: Phase 1 report 2000 http://www.csr.nih.gov/EVENTS/summary012000.htm accessed January 31, 2010

[B25] Bacchetti P Wolf LE Segal MR McCulloch CE Bacchetti et al. Respond to "Ethics and sample size - Another view" American Journal of Epidemiology 2005 161 113 113 10.1093/aje/kwi016 15632258

[B26] Bacchetti P Wolf LE Segal MR McCulloch CE Ethics and sample size American Journal of Epidemiology 2005 161 105 110 10.1093/aje/kwi014 15632258

[B27] Bacchetti P Wolf LE Segal MR McCulloch CE Re: "Ethics and sample size" - Reply American Journal of Epidemiology 2005 162 196 196 10.1093/aje/kwi177 15632258

[B28] Breslow N Are statistical contributions to medicine undervalued? Biometric Bulletin 19 1 2 http://www.tibs.org/WorkArea/showcontent.aspx?id=660 accessed January 31, 2010 10.1111/1541-0420.00001 12762435

[B29] Bacchetti P McCulloch CE Segal MR Simple, defensible sample sizes based on cost efficiency - Rejoinder Biometrics 2008 64 592 594 10.1111/j.1541-0420.2008.01004_5.x PMC2769573 18482055

[B30] Willan AR Optimal sample size determinations from an industry perspective based on the expected value of information Clinical Trials 2008 5 587 594 10.1177/1740774508098413 19029207

[B31] Willan AR Pinto EM The value of information and optimal clinical trial design Statistics in Medicine 2005 24 1791 1806 10.1002/sim.2069 15806619

[B32] Altman DG Moher D Schulz KF Peer review of statistics in medical research - Reporting power calculations is important British Medical Journal 2002 325 492 492 10.1136/bmj.325.7376.1364/a 12211233

[B33] Bacchetti P Peer review of statistics in medical research - Author's thoughts on power calculations British Medical Journal 2002 325 492 493

[B34] Senn SJ Power is indeed irrelevant in interpreting completed studies British Medical Journal 2002 325 1304 1304 10.1136/bmj.325.7375.1304 12458264 PMC1124761

[B35] Tukey JW Tightening the clinical-trial Controlled Clinical Trials 1993 14 266 285 10.1016/0197-2456(93)90225-3 8365193

