# Supplementary material for: Current sample size conventions: Flaws, harms, and alternatives
Source: BMC Med. 2010 Mar 22;8:17. doi: 10.1186/1741-7015-8-17 (PMC2856520; doi:10.1186/1741-7015-8-17)
Supplement: Additional file 1 — Comments on two possible objections. Discusses two possible objections to the case made in this paper. [file 1741-7015-8-17-S1.PDF]

Comments on two possible objections

My experience with peer reviews of previous related papers [1, 2], as well as published [3] and private correspondence about them, suggests that some readers may be quick to dismiss the case presented here because of perceived mistakes, poorly-thought-out counterarguments, or anticipated negative consequences of departures from current conventions. I comment here on two possible objections that seem particularly important, although this may only be a start on the objections that readers may formulate.

An initial reading of the threshold myth subsection may leave the impression that rejecting the myth depends on rejecting the established p-value threshold of 0.05, but this is not the case. I realize that the conventional $p<0.05$ threshold is widely accepted (despite controversy [4-6]), and most researchers have seen situations where a completed study just misses this and the investigators believe that a few more subjects would have resulted in “success” (i.e., $p<0.05$). This may seem like being on the wrong side of the threshold shown in Figure 1, but it does not imply that any threshold exists in a study’s projected value when it is being planned. Indeed, a mathematical argument has previously shown that rigidly accepting the $p=0.05$ threshold leads to projected value being determined by power [1], which has the shape shown by the solid line, not the mythical dashed line. Acceptance of the $p=0.05$ threshold therefore *contradicts* the existence of a threshold in pre-study projected value.

The design-use mismatch underlies an argument frequently used to support a requirement for high power: that $p<0.05$ in a study with low power is only weak evidence against the null hypothesis, because lower power implies that a higher proportion of $p<0.05$ results are type I errors (the null hypothesis is actually true) [7, 8]. This argument relies on using only the information that $p<0.05$, which would be a huge waste of a study’s other information if we were really concerned with evidence about the issue being studied; only in an automatic decision-making context would we ignore estimates and exact p-values. Examining the actual p-value obtained produces a different picture—a given p-value from a larger study indicates *weaker* evidence against the null hypothesis than the same p-value from a smaller study [9]. In the pure automatic decision-making context, sample size does not influence the rate or consequences of type I errors [10]; only type II errors are affected, and the influence of sample size on projected value has diminishing marginal returns as illustrated in Figure 1 [1].

References

1. Bacchetti P, McCulloch CE, Segal MR: **Simple, defensible sample sizes based on cost efficiency**. *Biometrics* 2008, **64**(2):577-585.
2. Bacchetti P, Wolf LE, Segal MR, McCulloch CE: **Ethics and sample size**. *American Journal of Epidemiology* 2005, **161**(2):105-110.
3. Halpern SD, Karlawish JHT, Berlin JA: **Re: "Ethics and sample size"**. *American Journal of Epidemiology* 2005, **162**(2):195-196.
4. Armstrong JS: **Significance tests harm progress in forecasting**. *Int J Forecast* 2007, **23**(2):321-327.
5. Cohen J: **The Earth is Round (p < .05)**. *American Psychologist* 1994, **49**(12):997-1003.

6. Goodman SN: **Toward evidence-based medical statistics. 1: The P value fallacy**. *Annals of Internal Medicine* 1999, **130**(12):995-1004.
7. O'Brien R: **Webinar 4: Classical sample-size analysis for hypothesis testing (Part II)**. [http://www.biopharmnet.com/doc/doc03002-05.html] 2009, accessed January 31, 2010.
8. Peto R, Pike MC, Armitage P, Breslow NE, Cox DR, Howard SV, Mantel N, McPherson K, Peto J, Smith PG: **Design and analysis of Randomized clinical-trials requiring prolonged observation of each patient .1. Introduction and design**. *British Journal of Cancer* 1976, **34**(6):585-612.
9. Royall RM: **The effect of sample-size on the meaning of significance tests**. *American Statistician* 1986, **40**(4):313-315.
10. Bacchetti P, McCulloch CE, Segal MR: **Simple, defensible sample sizes based on cost efficiency - Rejoinder**. *Biometrics* 2008, **64**(2):592-594.
